# Review of health research and data on/with racially minoritised groups: Implications for addressing racism and racial disparities in public health practice and policies in Europe: a study protocol

**DOI:** 10.12688/f1000research.128331.2

**Published:** 2023-12-07

**Authors:** Marie Meudec, Clara Affun-Adegbulu, Theo Cosaert

**Affiliations:** 1 The Population Data Science Hub, Department of Public Health, Institute of Tropical Medicine, Antwerp, 2000, Belgium

**Keywords:** racism, racial health disparities, racialised minority groups, health data, health research

## Abstract

Historically, across Europe, data and research on/with racially minoritised groups have not been collected or carried out in a sufficient, adequate, or appropriate manner. Yet, to understand emerging and existing health disparities among such groups, researchers and policymakers must obtain and use data to build evidence that informs decision-making and action on key structural and social determinants of health. This systematic search and review aims to contribute to closing this gap and promote a race-conscious approach to health research, strengthening the utilisation and deployment of data and research on/with racially minoritised groups in Europe. Its ultimate goal is to improve equality and equity in health*.

Concretely, the study will do so by reviewing and critically analysing the usage of the concepts of race, ethnicity, and their related euphemisms and proxies in health-related research. It will examine the collection, use, and deployment of data and research on/with racially minoritised groups in this area. The study will focus on Belgium, France, and the Netherlands, three countries with graphical proximity and several similarities, one of which is the limited attention that is given to racism and racial inequalities in health in research and policy. This choice is also justified by practical knowledge of the context and languages. The results of the review will be used to develop guidance on how to use and deploy data and research on/with racially minoritised groups.

The review is part of a larger project which aims to promote race-conscious research and data. The project does this by a three-pronged approach which: 1) highlights the need for a race-conscious approach when collecting and using data, carrying out research on/with racially minoritised groups; 2) builds expertise for their effective use and deployment, and; 3) creates a knowledge network and community of practice for public health researchers working in Europe.

## Introduction and rationale

While health disparities* ^,^ ^1^ linked to the socio-politically constructed concepts of race* ^,^ ^2^ and ethnicity* have long been established, the COVID-19 pandemic has brought renewed attention to the issue. Although most people have been affected by the pandemic, an increasing body of international research ^3^ shows that racially minoritised groups* have been disproportionately affected in terms of disease exposure, susceptibility to the disease, the severity of the disease and mortality rates. In addition to this, the measures taken to contain or mitigate the pandemic have had a particularly negative impact on the determinants of health and access to care for people within such groups. This has, in many cases, had negative consequences for their health statuses and health outcomes, which has ultimately further increased already existing health disparities between racially minoritised and majoritised groups ( Katikireddi *et al.,* 2021). Addressing this will require evidence-based decision-making and action on key structural and social determinants of health such as racism* and racial discrimination, which are mediated by race, ethnicity, and related concepts.

Yet, we start from the assumption that, in many countries across Europe, there is often inappropriate, inadequate, or insufficient use and deployment of data and research* on racially minoritised groups. The reasons for this assumption can be grouped into two main categories. One is the continuous emergence of biologically or genetically based race research which is often linked to scientific racism ( Roberts, 2011a, 2011b; Saini, 2019). Cerdeña, Plaisime, and Tsai (2020), recognising this, introduced the race-conscious approach* which, in contrast to the race-based approach*, focuses on racism and racial health disparities*. This race-conscious approach forms the basis and the goal of this project. The other category, which this project focuses on, includes issues related to the poor use and deployment of data and research on/with ^4^ racially minoritised groups, in the monitoring and tackling of health disparities, and public health policymaking and social change ( Farkas, 2017; Holtzman, Khoshkhoo, and Nsoesie, 2022).

Data and research on/with racially minoritised groups are often underutilised and under-deployed for three broad reasons. The first reason is related to data collection. In many countries, there is a lack of national data systems using race/ethnicity data, which means that there is limited statistical evidence on health disparities between racially minoritised and majoritised groups. This is partly due to national political models and philosophies around immigration and ethnic diversity management. Examples of this can be seen in the recent removal (law proposed in 2013 and accepted in 2018) of the word ‘race’ from the French Constitution ( Gay, 2015), and more broadly, in the republican model which is practised in France. According to this model, a recognition of ethnic and/or racial diversity is seen as subversive, and consequently, the collection of ethnic statistics for official purposes is deemed to be unnecessary or even contrary to the ideals of the model ( Rivenbark and Ichou, 2020). This can also be seen in Belgium where the Belgian Census does not include any questions on ethnicity, let alone racial identification (Lorant and Bhopal, 2011), or in the Netherlands, where the new population classification is based on the country of birth of the person, which implies a classification by origin and not by identification or experience of discrimination ( Statistics Netherlands, 2022). According to advocacy groups like the European Network Against Racism (ENAR) ^5^, this lack of data on ethnic/racialised groups makes it difficult to combat racism and racial inequalities, and it also means that clear guidelines with workable definitions on the subject matter are limited. This lack of (collection of) data at the individual/population level is often exacerbated by the lack of recognition and undervaluing of data such as lived experiences, testimonies, and other similar forms of knowing ^6^. Some of these problems could also be related to the lack of representation of racially minoritised groups at high levels of society. In addition to this, even in cases where data exists, and it is valued and recognised as such, it might still not be used for research to address racial health inequities, as many researchers do not have the opportunity and/or expertise to categorise, analyse data on racially minoritised groups, or to interpret and communicate the results in such a way as to promote racial health justice.

The second reason is linked to the research itself. There is disinclination among certain researchers to carry out any research linked to race and/or ethnicity, as the use of such variables in research continues to be seen as contentious, to some degree. Some researchers see these variables as valuable tools for analysing health inequalities* and addressing health inequities as well as the impact of various forms of racism (institutionalised, systemic/structural, interpersonal, internalised racism) on racially minoritised groups. Others, on the contrary, disagree, on the grounds that the data and results of research that use these variables can be misused and instrumentalised against racially minoritised groups. They also contend that such data and research can be used in ways that are sometimes difficult to anticipate, for instance to fuel stigmatisation and racial stereotyping, quoted as a “problematic use” of data by Nancy Krieger (2021). Additionally, research on/with racially minoritised groups is often seen as too difficult to implement in practice, due to challenges of finding appropriate approaches and solutions to conceptualisation, operationalisation, data collection, data management, data analysis, interpretation, representativeness, transferability and generalisability. For instance, in cases where data is collected on/with racially minoritised groups, it might be done in a “non-standardised” way which, certain researchers may find difficult or impossible to use in research ( Simon, 2005).

The third reason focuses on the results of research on/with racially minoritised groups. In many cases where data on racialised groups is available and it is being used for research, it does not necessarily lead to anti-racist interventions. Moreover, the results are frequently not used for advocacy, and/or the results and recommendations are not implemented or used to effect policy change and achieve real-life impact. This is amongst others because the results are deemed to be too specific, subjective, or politicised. Many researchers also consider transformative research, advocacy and activism to be beyond their mandate. Furthermore, similarly to broader society, within the field of health, there are strong tendencies to erase contributions from marginalised/racialised researchers or to erase race when analysing inequalities, as was for example the case with the concept of intersectionality* in feminist studies ( Bilge, 2013). In contrast, when the results are used in research or policymaking, this is generally done either through (un)conscious racial biases in the framing of social problems, or without sufficient attention to unintended consequences which means that in some cases, results, instead of supporting efforts to address racial health disparities, actually (inadvertently) reinforce stereotypes about ethnic/racial groups ( Kaplan and Bennet, 2003; Zuberi, 2003; Laveist, 1996).

The consequence of all this for public health is the adoption and implementation of policies that are intended to be race-neutral but which in fact produce a colourblind paradigm that reproduces ‘methodological whiteness’ ^7^ and creates or exacerbates health inequities* and inequalities amongst racially minoritised groups. This criticism is reflected in the widespread calls to decolonise health research, improve equality and equity in health in an intersectional way, and ultimately, achieve social and racial justice. Decolonial approaches to global and public health* have been a growing field in recent years and offering many opportunities for collaboration with anti-racist public health and critical race theories ( Meghji and Niang, 2022). These calls are being responded to at all levels of society. In 2018, for instance, there was a 65% increase in the number of English articles that were published on racism in healthcare at the global level ( Hamed *et al.,* 2022). In the same year, the European Union (EU) High Level Group on Non-discrimination, Equality, and Diversity ^8^ adopted a set of non-binding guidelines on how to improve the collection and use of equality data, compiled practices implemented at national level related to the set of guidelines and developed a diagnostic tool/checklist with which to assess the availability and quality of equality data collected at national level ^9^.

This project likewise aims to contribute to improving equality and equity in health, by promoting a race-conscious approach to health research and strengthening the utilisation and deployment of data and research on/with racially minoritised groups. We do so by taking a three-pronged approach which highlights the need for a race-conscious approach while using data and research on/with racially minoritised groups; builds expertise for their effective utilisation and deployment; and creates a knowledge network and community of practice for public health researchers working in Europe.

## Research steps

The project begins with a literature review which critically analyses the way race, ethnicity, and related terminology euphemisms and proxies* are conceptualised, operationalised, and used in public health research in three countries in continental Europe. It then goes on to critically examine, using literature from other countries or other research fields on this issue, how research on racialised minority groups is conducted. The results will then be used to develop guidance on how to utilise and deploy data and research on racialised minority groups. Finally, as a follow-up to the review, our findings will be stored in a knowledge repository that is accessible to health researchers (see the project’s research steps in Table 1 below). The overarching goal is to contribute to addressing health disparities among racialised minority groups, across Europe.

**Table 1.** RECoRD project, the research steps.

RECoRD project Race-Conscious Research and Data *		
Review of health research and data on/with racially minoritised groups	Systematic search	2022
	Critical analysis	2022-2023
Guidance on how to use and deploy data and research on/with racially minoritised groups	Proposed guidance	2023
	Participatory development of final guidance	2023-2024

* See key definitions section.

### Key definitions

1. Race: Refers to socially and politically constructed perceptions of differences among people based on phenotypic characteristics such as skin colour. Although the sciences have been (and still are) heavily involved in the production of race and racial categorisations, there is no scientifically supported biological basis for racial categorisation. However, various societal actors construct races as real, which has a variety of detrimental implications for economic, political, social, and cultural life ( CIHI, 2022).
2. Ethnicity: A multi-dimensional social construct based on cultural distinctiveness and shared group cultural identity and characteristics. Examples of ethnic characteristics are, amongst others, language, and cultural norms, which are sometimes linked to religion and nationality. Different from, but often used as a euphemism or proxy for race ( Song, 2018; CIHI, 2022).
3. Racial and ethnic euphemisms and proxies: A word or expression used inappropriately or inadequately to designate and describe racially minoritised groups. We understand concepts as racial and ethnic euphemisms and proxies when there is a clear mismatch between 1) the used concepts or variables and 2) the aim of the research or the interpretation of the results. This includes the use of terms which are related to concepts like migration, religion, language, origin, postcode (= place-based discrimination), citizenship, nationality, and culture. Note however that the concepts mentioned above are not inherently proxies or euphemisms for race and ethnicity, ^10^ and can be of adequate use depending on the topic of research.
4. Racialisation: A complex, contradictory and arbitrary process through which groups and individuals are assigned a particular ‘race’ and on that basis subjected to differential and/or unequal treatment. Put simply, racialisation is “the process of manufacturing and utilising the notion of race in any capacity” ( Dalal, 2002, p. 27). While white people are also racialised, this process is often rendered invisible or normative to those designated as white. As a result, white people may not see themselves as part of a race but still maintain the authority to name and racialise ‘others’. The individual and group identities of members of racialised groups shape both their relationships and interactions between each other, as well as with members of the out-group, and it influences social practice, and engagements with time, space, social structures and institutional systems. Racialisation thus has impacts on every aspect of life ^11^.
5. Racially (minoritised) groups: Refers to groups that are subject to racialisation and are also minoritised, marginalised or underrepresented based on various characteristics such as skin colour, migration status, citizenship, religion, culture, language or geographic location ^12^. To emphasise the process of racialisation, some authors use either this wording ( Milner and Jumbe, 2020; Rai *et al.*, 2022) or “marginalised racial groups” ( Barber, 2020). This quote from Selvarajah *et al.* (2020: 2-3) is particularly interesting for a reflection on the term ‘minoritised’ ^13^: “We recommend the term minoritised, which emphasises active processes, shifting beyond binary discussion of minority versus majority. We build on existing explanations to define minoritised, as ‘individuals and populations, including numerical majorities, whose collective cultural, economic, political and social power has been eroded through the targeting of identity in active processes that sustain structures of hegemony.’ Power is emphasised as central to racism and intersecting forms of discrimination. It highlights maintenance of structures which diminish minoritised people’s capability to lead healthy lives. It neither singles out nor creates groups, and adds more nuance than words like marginalised by connecting back to terms such as ethnic minority, thus acknowledging existing literature while resisting its coupling with dubious assumptions about ethnicity. It is important to acknowledge the fact that racially minoritised groups are not homogenous groups, given the intersecting forms of oppression within minoritised groups. Although we clearly want to avoid any form of oversimplification, and we acknowledge the fact that health inequities cannot be reduced to race alone, we see a stark omission of research analysing racism in health in Europe; therefore, we see this one-dimensional study as a first essential step to enable the development of more complex research using intersectional lens in the future.”
6. Racism: Organised systems within societies that cause avoidable and unfair inequalities in power, resources, capacities, and opportunities for racially minoritised groups ( Paradies, Ben, Denson *et al., *2015). Racism can manifest through beliefs, stereotypes, prejudices, or discrimination. This encompasses everything from open threats and insults to phenomena deeply embedded in social systems and structures. Racism can occur at multiple levels, including internalised (the incorporation of racist attitudes, beliefs or ideologies into one’s worldview), interpersonal (interactions between individuals) and systemic (for example, the racist control of and access to labour, material and symbolic resources within a society) ( Paradies, Ben, Denson *et al., *2015: 2).
7. Health equity: Refers to the absence of differences in health associated with social disadvantages that are modifiable, and considered unfair. This means everyone has a fair chance to reach their full health potential without being disadvantaged by social, economic and environmental conditions ( CIHI, 2022; NCCDH, 2014).
8. Health disparities or health inequalities: Refers to a condition in which different social groups have different health outcomes. Generally, disadvantaged social groups such as the poor, racially/ethnic minoritised groups, women and other groups who have persistently experienced social disadvantage or discrimination systematically, experience worse health or greater health risks than more advantaged social groups ( Braveman *et al.*, 2004; Braveman, 2007). When systemic barriers to good health are avoidable yet still remain, they are often referred to as ‘health inequities’. ^14^
9. Racial health disparities: Refers to health disparities that exist between racially minoritised groups and the racially majoritised group, as compared to the racial majority. It describes the increased presence and severity of certain diseases, poorer health outcomes, and greater difficulty in obtaining healthcare services. Usually, it is the racially minoritised groups who are at a disadvantage compared to the racially majoritised one.
10. Intersectionality: The complex, cumulative way in which the effects of multiple forms of discrimination (such as racism, sexism, classism, xenophobia, and religious discrimination) combine, overlap, or intersect especially in the experiences of marginalized individuals or groups ( Crenshaw, 1989).
11. Race-based approach: Based on a biologically essentialist conception of race according to which all members of a racial category are believed to have defined shared physical or genetic characteristics, or a specific biological essence. This assumption allows the members of the group to be seen, both by themselves and by others, not as individuals with personal traits, but rather as prototypes of the collective with identical traits and characteristics, which leads to stereotyping, essentialization, fixity, homogenisation.
12. Race-conscious approach: Focuses on racial discrimination and racism as central issues, in contrast to the race-based approach. As a reference point, Cerdeña, Plaisime and Tsai (2020) introduce “race-conscious medicine as an alternative approach that emphasises racism, rather than race, as a key determinant of illness and health, encouraging providers to focus only on the most relevant data to mitigate health inequities”.
13. Race-conscious research and data: Following the previous definition, we would like to define ‘race-conscious research and data’ as an area of research that aims to address racial inequities in health, combat racism in healthcare and promote racial justice in health.
14. Racial justice in health: Using the race-conscious approach to tackle racial health disparities and injustices and improve health among racially minoritised groups in an intersectional way, with the aim of advancing equality and equity in health and ultimately, achieving social and racial justice ^15^.
15. Decolonial approaches to Global and Public Health: This field is not homogeneous but decolonising global and public health implies re-politicising and re-historicising health at all levels: epistemic and theoretical (production and distribution of knowledge), ontological, educational, organisational, healthcare-related, etc. ( Bhakuni and Abimbola, 2021; Affun-Adegbulu and Adegbulu, 2020; Büyüm *et al.,* 2020; Naidu, 2021).
16. Data: Any type of information that is collected to be examined, considered, and used for research as well as to support decision-making. This includes quantitative information, such as measurements and calculations, and qualitative information, such as lived experiences and blog posts ^16^.
17. Research: “A detailed study of a subject, especially in order to discover (new) information or reach a (new) understanding ^17^”, which involves “weaving together different strands of information, thought, and data ^18^”, amongst others, to contextualise both the research and its findings. As social scientists and public health researchers, we see research as an activity that leads directly to practical applications and engagement/advocacy in the field of health.
18. Othering: Processes of distancing and differentiation in which certain individuals, groups or practices are defined and labelled as ‘Others’, thus not corresponding to the norms of a social group. It refers to a binary conception of ‘us/them’, usually involving stereotypes of ‘them’ and hierarchical power relations, including practices of inclusion and exclusion. See for instance Udah (2019).
19. Xenophobia: “Attitudes, prejudices and behaviour that reject, exclude and often vilify persons, based on the perception that they are outsiders or foreigners to the community, society or national identity.” (European Commission, Migration and Home Affairs) As such, xenophobia needs to be distinguished from racism, which concerns (systemic) acts of discrimination towards someone based on their perceived affiliation to a racially minoritised, regardless whether this person is seen as a foreigner or not ( Suleman *et al.,* 2018: 2018). This distinction is important, as the mechanisms and outcomes of both forms of discrimination are different and can affect different groups.

### Objectives

The objectives of the literature review are to:

1. Examine how data on racially minoritised groups is used (= conceived, collected, analysed, interpreted, reported) in health research
2. Examine the ways in which this data is used to address racial health inequities
3. Critically analyse the way race, ethnicity and related euphemisms and proxies are conceptualised, operationalised, and used in health research
4. Develop guidance on how to appropriately utilise and deploy data on/with racially minoritised groups, how to undertake race-conscious research and how to effectively use the results to address racial health disparities

### Scope of the research

Thematic scope: The review will take race and ethnicity in health research as a focus of analysis. It will expand to include related euphemisms and proxies such as migration, citizenship, nationality, religion, culture, language, postcodes, etc. In the analysis, we will consider other characteristics which influence and shape health inequities, such as gender, sexuality, disability, age, socio-economic condition, and geographic location. This will ensure that we integrate relevant intersecting determinants of health inequalities and inequities in our analysis of health disparities among racialised groups ( Smedley *et al.*, 2003). It will also allow us to draw attention to the complexities of vulnerabilisation, its different forms and its various causes as well as the interplay between them.

Geographical context: The review will focus on research on Belgium, France and the Netherlands (and their overseas territories), three countries in continental Europe which have been selected for their geographical proximity, as well as their linguistic and cultural similarities and differences. In addition to this, given the personal and professional background of the review team members, the team has an in-depth knowledge of these three countries. Three countries were chosen for the study, for practical reasons, as resources constraints mean that we do not currently have the ability to conduct a Europe-wide study. We however hope to be able to both deepen this work and extend it to other European countries in a second phase.

Timespan: The review will cover the period between 2018 and 2022 which will allow us to take into account data and research on/with racially minoritised groups from before and during the COVID-19 pandemic (two years before, two years during). This is because, as argued above, the COVID-19 pandemic has led to an increased focus and attention on the issue of health disparities between racially minoritised and majoritised groups.

## Methods

A systematic search and review approach will be taken to this review, as this combines strengths of a critical review with those of an exhaustive search process. This approach is especially suited for our review because by facilitating the comprehensive exploration of what is known about the topic, it supports the synthesis of best evidence and the generation of recommendations for practice ( Grant and Booth, 2009).

### Review questions

The questions which will guide the review are as follows:

1. What terminology is used for health research on/with racially minoritised groups, and how are they operationalised?
2. What type of data on race, ethnicity and related euphemisms and proxies is used, and why?
3. How is research on/with racially minoritised groups carried out?
4. What evidence is available on the use of racially minoritised groups data to promote racial equity in health?
5. What are best practices on research and the use of data on/with racially minoritised groups, and why?

### Data collection

**Databases**

The databases listed below will be used in this review. They were chosen for their large collections of both peer-reviewed and grey literature, which will ensure that we can capture the variety of published information on the subject matter.

• PubMed: https://pubmed.ncbi.nlm.nih.gov/
• Scopus: https://www.scopus.com/home.uri
• Web of Science: www.webofscience.com
• Cochrane Library: https://www.cochranelibrary.com/central/about-central

**Search strategy**

The search strategy was developed by creating a list of search terms that are relevant to the research questions and combining them as follows:

(race OR racial* OR ethnic* OR cultur* OR language OR linguistic OR religio* OR migra* OR immigrant OR foreign* OR “third country national” OR allochthonous OR residen* OR undocumented OR illegal OR irregular OR refugee OR asylum OR nationality OR citizen OR “non-citizen” OR minorit* OR gyps* OR roma OR traveller OR ancestry OR “family background” OR heritage OR origin OR neighborhood OR neigbourhood OR “postal code” OR postcode OR marginalised OR marginalized OR vulnerable OR precarious OR communit* OR “population group”)

AND

(“health”)

AND

(Belgium OR “Netherlands” OR France)

Search strings will be created from these terms and adapted to the requirements of each database. Given that the search strategy and this protocol was developed in the very early stages of the review process, we see the above as a non-exhaustive list. In addition to conducting explorative searches to refine the strategy, therefore, we will also take an inductive approach, in which we allow concepts that emerge from the review to further inform the search strategy.

The results of the search strategy will be refined by language and date to include only publications that were written in English, French, and Dutch and which were published from January 1 ^st^, 2018 until July 8 ^th^, 2022.

The database searches will be supplemented by reference mining of the selected publications to ensure that relevant documents or articles that might have been missed, are identified and included. In addition, purposive manual searching of websites of key actors and organisations will be carried out to identify relevant grey literature we might have missed in the database searches.

The details of the search process, as well as the results of the searches conducted will be documented as meticulously as possible, in order to maximise recall and ensure that the process can be reported and reproduced accurately.

**Selection process**

The citations produced by the search strategy will be screened for relevance and for inclusion in the study. To be eligible, the article or report must have both health AND race, ethnicity, or related concepts as its subject matter.

The research will be done by a core team of three researchers, who will be supported periodically by three master students with relevant experience and knowledge.

In the first instance, two researchers, in consultation with the third researcher, will search the selected databases for relevant citations, using the developed search string. The results of this search procedure will then be uploaded into Covidence, a systematic review management software which supports some of the steps of the review process.

Next, Covidence will be used to identify and automatically remove duplicates, a process that will be verified by one researcher. Given that Covidence is limited in its ability to recognise duplicates, the selected references will be exported to Zotero by one researcher, who will then do an additional duplicate check.

After this, the title and abstract screening of the documents in Covidence will be done by three students, who are supported and supervised by one researcher. From this stage onwards, weekly discussions will be held to streamline and systematise the selection process as much as possible. Following this initial selection, the full texts of the selected documents will be obtained and checked meticulously against the review’s inclusion and exclusion criteria. This full text screening will be done in Covidence by the three researchers and three students, with every document being checked at least twice to minimise bias and error. The process will be set and carried out in such a way as to ensure that each full text is screened by at least one of the three researchers from the core team. Conflicts will be discussed and resolved as a group, during the weekly meetings, and potential deviations from the review protocol will be documented and reported.

The types of documents to be included are peer-reviewed primary studies and reviews; preprints; commentaries; editorials, published in a scientific journal and of which a full-text version is available. In addition to this, we will also include published grey literature where the full text is available online.

**Data extraction**

Once the screening process is finished, the data on the study characteristics and other relevant variables will be extracted by the three researchers and one student from the final collection of retained documents, in a systematic way. This will be done in Covidence and the extracted data will be stored in Excel. In order to minimise error, the team will use a standardised extraction sheet that has been designed collaboratively by the three researchers and the three students, with some input from the extended project group ( Meudec *et al.,* 2022c).

Approximately 30 variables will be extracted from the publications that are included in the review. This will include information on the:

1. Study characteristics
  a. Publication (title, year of publication, author(s) and their affiliation, journal, type of document)
2. Variables of interest
  a. Concepts that are used for health research on/with racially minoritised groups and how they are operationalised
  b. Research methodology and methods used
  c. The data used, and how this is collected, and applied

A full overview of the variables to be extracted can be found in the Data Extraction sheet (see Data availability).

### Data management

Citations generated from the search strategy will be reviewed using the Covidence software which will be used to identify publications for inclusion in the review. These will then be uploaded and stored in a Zotero library. The data extraction of selected publications will be done using Covidence.

### Data analysis

First, a descriptive analysis will be done to provide an overview of the data that is extracted from the included publications, using quantitative and qualitative methods.

Following this, a critical analysis will be undertaken to identify the:

1. Concepts that are used for health research on/with racially minoritised groups
2. Types of data on race, ethnicity and related euphemisms and proxies that are used, and arguments put forward to justify their use
3. Methodology and methods that are used for research on/with racially minoritised groups, with a particular focus on recommendations, research gaps, innovative approaches, and methods

The results of the critical analysis will then be used to inform the development of proposed guidelines for best practices in the use and deployment of data and research in racialised minority groups, with the aim of addressing health disparities.

### Reporting and registration

The first and final drafts of the review protocol are stored on a community platform on Zenodo ( Meudec *et al., *2022a, 2022b). This protocol has been completed in line with the PRISMA-P reporting guidelines ( Meudec *et al., *2022d).

The finalised review protocol will be registered and peer-reviewed on the Open Research Platform F1000Research.

### Review team

**Core team**: Marie Meudec, Clara Affun-Adegbulu, Theo Cosaert

**Review team**: Marie Meudec, Clara Affun-Adegbulu, Theo Cosaert, Eskedar Getie Mekonnen, Lidvine Ngonseu Harpi, Enata Mushimiyimana

**Extended project group**: Soledad Colombe, Charles Ddungu, Sarah Demart, Cleo Maerivoet, Lazare Manirankunda, Joris Michielsen, Claudia Nieto, Christiana Nöstlinger, Jef Vanhamel, Ella Van Landeghem, Tine Verdonck

Following ITM guidelines on authorship ( Institute of Tropical Medicine, 2017), the core team has carried out the following tasks: 1) conception of the work; 2) design of the study and drafting of the review protocol; 3) execution of the study; 4) data analysis; 5) data interpretation; 6) writing of the review. The review team will participate in tasks 2, 3, 4, 5, 6. The extended group has been, and will be involved in steps 2, 5, and 6.

### Positionality

Marie Meudec is a white researcher who has no personal experience of racism. From personal and professional experience - a) research on health inequalities and discrimination based on gender, sexuality, migratory status, different forms of spatial marginalisation, police racism, etc, b) providing expert court testimony on police racial profiling in Canada and asylum cases in the UK; and c) organising and facilitating anti-racism workshops on whiteness and white supremacy in Canada, Marie has developed a sensitivity to issues of racism and racial justice in the countries where she has lived and worked (France, Canada, UK, Haiti, Belgium).

Clara Affun-Adegbulu is a Black woman with a lived and personal experience of anti-Black racism and misogynoir, amongst others. During her nursing studies and throughout her career as a district and psychiatric nurse in Belgium, France and the UK, Clara also gained direct professional experience of anti-Black racism, race- and ethnicity-based discrimination more generally, as well as the intersections of the two with other forms of discrimination. Her understanding of, and sensitivity to these issues has further developed, as a result of her work as a public health researcher studying health equity, including among migrants and displaced populations from fragile and conflict-affected settings.

Theo Cosaert is a white male junior researcher with no personal experiences of racism. He grew up and was trained in a West-European context (Belgium and the UK) and his academic practice is shaped by these schools of thought. He was trained in sociology and in medical anthropology, and all of his previous work focused on experiences of and barriers to the healthcare system. He tries to centralise perspectives of minoritised groups in his research by listening and by creating space where and when he can do so.

### Data availability

The list of references used in the review will be stored on Zotero, while the project documents will be stored on Zenodo, as well as the Data Science Hub/ITM website. Both the review references and project documents will be open access and freely accessible to the public ( Meudec *et al., *2022a, 2022b, 2022c, 2022d).

### Dissemination

The finalised review protocol will be shared online on the F1000research website. An overview of the output of the RECoRD project can be found here https://linktr.ee/record_itm. The review results have been and will be shared and discussed during conferences (AfroEuropeans Conference Sept 2022; European Public Health Conference Nov 2022; Be-Cause health conference 2023; ECTMIH 2023) and seminars at the Institute of Tropical Medicine (Belgium). The review results will also be submitted to an open access scientific journal after finalisation.

### Outcomes and prioritisation

The primary outcome will be a list of concepts (related to race, ethnicity and their related euphemisms and proxies) that are used in health research on/with racially minoritised groups. This list will also include – if provided – the definitions and justifications for such a use, and the ways these concepts are operationalised in research.

A secondary outcome will focus on the use of such concepts (context, research questions, research methodologies, results).

A third outcome will examine the recommendations, research gaps, and innovative approaches.

A fourth outcome will consist in the development of proposed guidelines for best practices in the use and deployment of data and research on/with racially minoritised groups, with the aim of addressing health disparities.

The results of this review will be developed in a manuscript submitted to a scientific journal for publication.

### Risk of bias

In addition to asserting our respective positionalities, we also identify several biases and limitations in our work. Firstly, our core team is small and represents only a margin of the diverse intersections of social identities that are present in society. Specifically, the core team is mostly trained in North American and European higher education, which implies that our own frames of reference are primarily Western. The categories and concepts we employ are therefore partial and limited, and inevitably bias our questions, methods and the interpretation and analysis of data both consciously and unconsciously. We are trying to mitigate this bias continually, for example by drawing from the field of Critical Race Theory, by gathering feedback from a larger and more diverse team of researchers, and by holding meetings with a range of stakeholders during future stages of the research (analysis, writing, and guideline development).

Second, this research project focuses on ethnic and racial disparities in health, and thus centralises/emphasises race and racism. This may be a bias in that, by focusing on racial disparities, we temporarily sideline other criteria present within intersecting systems of oppression. We see this research as a first step in demonstrating the lack of a racial lens in health research, and we know that future research will need to take an intersectional lens as its starting point.

A further bias may arise from the fact that we only have access to published results, and we do not have access to all the internal discussions or specific issues related to the use of specific terms or concepts over other options. As this analysis is something of a retrospective study, our analysis is based solely on the information that the authors decided to incorporate in their published manuscripts. This constitutes a bias in the sense that we may risk attributing certain intentions to the authors even though we cannot confirm this in this review. We regret the fact that, in general, authors of scientific articles in this field are not more explicit in justifying their use of a particular terminology.

Another bias could stem from our personal and professional involvement with systemic racism in health. Given our respective positionalities and lived experiences, and given that we do not ultimately aim to achieve objectivity or neutrality in this research, we would like to acknowledge that our judgment in analysing research papers can sometimes be harsh, especially after analysing several documents containing racial/ethnic proxies and euphemisms in the course of a single day. We try to mitigate this by holding weekly meetings with the core team, during which we share our emotions and discuss elements that emerge in the course of the research.

Finally, this research project focuses on systemic racism in a European context. As such, health disparities are conceptualised in such a way that the white racial majority is seen as the benchmark of good health from which racially minoritised groups diverge. While we recognise that this is a simplified and binary way of analysing disparities, we also want to acknowledge that in doing so we are using whiteness as the default. We are aware of the need to decenter whiteness in research, and we urge the reader to be aware of this bias. Here are a few suggestions of intellectual traditions centering the voices of racialised groups that you can get inspiration from: Black Feminist Thought, Black Intellectual Tradition, Critical Race Theory, Indigenous methodologies, etc.

## Data Availability

No data are associated with this article. Zenodo: Review protocol - First draft - Review of health research and data on racialised minorities: Implications for addressing racism and and racial disparities in public health practice and policies in Europe. https://doi.org/10.5281/zenodo.7155891. ( Meudec *et al.*, 2022a). This project contains the following extended data:
• First draft of the review protocol, October 7, 2022. Data are available under the terms of the Creative Commons Zero “No rights reserved” data waiver (CC0 1.0 Public domain dedication). Zenodo: Review protocol - Final version - Review of health research and data on racialised minorities: Implications for addressing racism and and racial disparities in public health practice and policies in Europe. https://doi.org/10.5281/zenodo.7298547. ( Meudec *et al., *2022b). This project contains the following extended data:
• RECoRD Review protocol_final version.pdf. (Final version of the review protocol, November 7, 2022). Data are available under the terms of the Creative Commons Zero “No rights reserved” data waiver (CC0 1.0 Public domain dedication). Zenodo: Data Extraction Sheet for the Review - 2. https://doi.org/10.5281/zenodo.7473314. ( Meudec *et al., *2022c). This project contains the following extended data:
• Data extraction sheet.pdf (Data extraction sheet using approximately 30 variables - study characteristics and various variables of interest). Data are available under the terms of the Creative Commons Zero “No rights reserved” data waiver (CC0 1.0 Public domain dedication). Zenodo: PRISMA-P checklist for ‘Review of health research and data on racialised groups: Implications for addressing racism and racial disparities in public health practice and policies in Europe - Study protocol’. https://doi.org/10.5281/zenodo.7458371. ( Meudec *et al., * 2022d). Data are available under the terms of the Creative Commons Zero “No rights reserved” data waiver (CC0 1.0 Public domain dedication).

## Footnotes

^1^
All terms followed by an asterisk (*) are defined in the ‘Key definitions’ section of this protocol.

^2^
The authors follow the widely accepted notion that race has no biological basis. Regardless, race exists as a social reality and thus has real consequences. See also: key definitions.

^3^
For international literature, see for instance Aldridge *et al. *(2020), Baqui *et al. *(2020), Linos *et al. *(2022), Miconi *et al. *(2021), Mukumbang *et al. * (2020), OECD (2020), Poteat *et al. * (2020), Van Dorn *et al. * (2020), Wadhera *et al. *(2020), Williamson *et al. *(2020), Yaya *et al. *(2020). For Belgium, see Vanthomme *et al.* (2021), for France, see Carillon *et al. *(2020); and for the Netherlands, see Coyer *et al.* (2021).

^4^
The term ‘on/with’ is used deliberately. On the one hand, we want to acknowledge that in reality, much research is not done ‘with’ the communities it focuses on. On the other hand, however, we do not wish to reify and normalise the formulation ‘doing research on’ and the associated academic culture throughout our text. In addition, we only have access to the data that researchers have chosen to publish, so we do not always know whether the research is actually done ‘on’ or done ‘with’ the community in question. For all these reasons, using the formulation ‘on/with’ seems to be the most nuanced and correct way.

^5^
See https://www.euractiv.com/section/non-discrimination/news/unbalanced-hate-lack-of-data-abets-geography-of-discrimination-in-europe/ (accessed August 26th, 2022).

^6^
For an illustration, see this scoping review on empirical studies published in Scandinavian Journal of Public Health ( Elstad *et al., *2022) or see the epistemological discussion made by Lisa Bowleg on the uses of qualitative methodologies in Critical Health Equity Research ( Bowleg, 2017).

^7^
See Bhambra (2017a, 2017b). “‘Methodological whiteness’, I suggest, is a way of reflecting on the world that fails to acknowledge the role played by race in the very structuring of that world, and of the ways in which knowledge is constructed and legitimated within it. It fails to recognise the dominance of ‘whiteness’ as anything other than the standard state of affairs and treats a limited perspective – that deriving from white experience – as a universal perspective. At the same time, it treats other perspectives as forms of identity politics explicable within its own universal (but parochial and lesser than its own supposedly universal) understandings.” (2017b).

^8^
See https://ec.europa.eu/transparency/regexpert/index.cfm?do=groupDetail.groupDetail&groupID=3328

^9^
See the Guidance note on the collection and use of equality data based on racial or ethnic origin, accessed online Aug. 26, 2022: https://ec.europa.eu/info/sites/default/files/guidance_note_on_the_collection_and_use_of_equality_data_based_on_racial_or_ethnic_origin.pdf

^10^
It has to be noted here that the concept of ethnicity is itself sometimes used as a proxy to not talk about race ( Song, 2018).

^11^
Inspired by SOURCE: Alberta Civil Liberties Research Centre, “Racialization” (2018)/Calgary Anti-Racism Education, “CARED Glossary” (2020).

^12^
See for instance a definition proposed by Souissi (2022) on the Canadian Encyclopedia. Access online, September 1st, 2022: https://www.thecanadianencyclopedia.ca/en/article/racialized-minorities

^13^
We also refer readers to these writings by Gunaratnam (2013) for an important reflection on the term ‘minoritised’.

^14^
What Is Health Inequity?, see https://www.vdh.virginia.gov/health-equity/unnatural-causes-is-inequality-making-us-sick/what-is-health-inequity/#:~:text=%E2%80%9CDifferences%20in%20health%20status%20among,living%20in%20various%20geographic%20localities.%E2%80%9D (site visited June 22, 2022).

^15^
Inspired by Reference to the Praxis Project,: see health law as social justice, 2014.

^16^
Inspired by https://dictionary.cambridge.org/dictionary/english/data

^17^
See https://dictionary.cambridge.org/dictionary/english/research

^18^
See https://blog.scienceopen.com/2016/05/why-context-is-important-for-research/
