# Supplementary material for: Review of health research and data on/with racially minoritised groups: Implications for addressing racism and racial disparities in public health practice and policies in Europe: a study protocol
Source: F1000Res. 2023 Dec 7;12:57. Originally published 2023 Jan 13. [Version 2] doi: 10.12688/f1000research.128331.2 (PMC10904934; doi:10.12688/f1000research.128331.2)
Supplement: Peer review history

# Reviewer report: Reviewer response for version 2

**DOI:** 10.5256/f1000research.159496.r228518

**Published:** 2024-02-15

**Authors:** Elie Azria

**Affiliations:** 1 INSERM, Université de Paris, Paris, France

Although I do not fully agree with some of the authors' responses, and the fact that the project has not been registered on the PROSPERO platform is a limitation of the project (which may also limit access to high-impact journals for publication), I consider the revisions to be appropriate and wish the project team all the best.

Is the study design appropriate for the research question?

Partly

Is the rationale for, and objectives of, the study clearly described?

Yes

Are sufficient details of the methods provided to allow replication by others?

Yes

Are the datasets clearly presented in a useable and accessible format?

Not applicable

Reviewer Expertise:

NA

I confirm that I have read this submission and believe that I have an appropriate level of expertise to confirm that it is of an acceptable scientific standard.

# Reviewer report: Reviewer response for version 2

**DOI:** 10.5256/f1000research.159496.r228519

**Published:** 2024-02-09

**Authors:** Chinelo L. Njaka

**Affiliations:** 1 Goldsmiths University of London, London, England, UK

***Race, Health, and Inequality in Europe: A Study Protocol Review of “Review of Health Research and Data on Racialised Groups: Implications for Addressing Racism and Racial Disparities in Public Health Practice and Policies in Europe,” Version Two.***

**Introduction:**

 As overviewed in my previous review of “Review of Health Research and Data on Racialised Groups: Implications for Addressing Racism and Racial Disparities in Public Health Practice and Policies in Europe,” a broad range of actors from non-governmental organisations, academic institutions, and elsewhere have been advocating for the disaggregation of race data—particularly in the European context. The lack of doing so obfuscates key factors in health and other disparities, and by collecting data on race, these disparities can be illuminated and combatted through informed and evidence-based policy making ( UN OHCHR 2018; see also previous review for more details).

 The current version of the paper shows strengthened improvement and refinement from the previous version, offering an important contribution to existing scholarship around race and racialisation in the European Union, the socio-political reasons behind the lack of disaggregated race data collection, and an important challenge to that status quo through the systematic review of current literature that reveals the real challenges in health—and more broadly—that exist and persist through ‘race neutral’ policies.

**Study Protocol Assessment:**

 The significance of this study protocol remains in its potential to inform and shape ‘race-conscious’ approaches to future public health policies that are able to acknowledge and address racialised disparities in The Netherlands, Belgium, and France specifically, as well as Europe more broadly.  See my previous review for further detailed assessment, which all remains relevant to the current version of the protocol.

**Areas for improvement and Recommendations:**

 The protocol has been greatly improved in the current version, though there remain a few minor areas of note that could be addressed to strengthen the protocol further.

 The use of the word ‘assumption’ to describe the starting point for the authors does a disservice to the premise of the study. I understand that this was a change in response to the comments of another reviewer, but I do not think mere ‘assumption’ is accurate. There has been much documented challenge and scholarly enquiry around race (dis)aggregation in data collection over decades, so adding references to the protocol will support the authors’ starting position as more than ‘assumption’ and better contextualise the current situation and issues with it, especially for those who are unaware or have otherwise not identified any issues with ‘race-neutral’ policies.

 I agree that moving towards research that works *with* racially minoritised individuals and groups, rather than only *on* them is an important way forward in collecting data and improving research efficacy in general. However, as the authors noted, that is not yet happening frequently, and they are unsure if this has happened (and to what extent) in the research being analysed. Thus, the use of ‘on/with’ as aspirational, rather than as an accurate description is insufficient and potentially problematic because it can be misleading. Perhaps think through an alternative way to convey this current tension, as it is an important one with which to grapple and I commend the authors for this consideration.

 The working definition for ‘race’ is not detailed sufficiently. The concept of race—and therefore the process of racialisation—is not limited to skin colour, so currently the definition reads more as one for ‘colourism.’ Providing additional examples of phenotypic characteristics (and/or others outside of phenotype) that can contribute to conceptions of race would improve this greatly.

 For ‘ethnicity,’ the working definition could also be improved upon, as currently it reads as being synonymous to ‘culture.’ Editing to make clear that ‘ethnicity’ is distinct from ‘culture’ will not only increase the clarity for the readers, but also may help clarify what the researchers will be looking for during their literature review. If ‘culture’ will also be salient, which it likely will be, the authors might want to consider adding a separate definition for ‘culture’ to the protocol, as well.

 The term ‘health inequities’ is introduced twice on page 4 before an asterisk on page 5 is used to signal that there is a definition below. However, there is not a definition given for this particular term. Previously, I suggested defining terms in the positive (e.g., ‘equity’) versus negative (e.g., ‘inequity’) for clarity, and I see that this recommendation was partially implemented. In light of the revisions made and definitions given, how is ‘equity’ distinct from ‘equality’? The definition given for ‘equity’ reads as definition for ‘equality.’ It would strengthen the protocol greatly to provide specific definitions for each concept in a clear and specific way that reflects how they are being operationalised (distinctively) in the research. At present, the definitions given leave unclear what ‘(health) equality’ is (and how it is distinct from ‘[health] equity’) and what ‘(health) inequity’ is (and how it is distinct from ‘[health inequality’).

 Lastly and minorly, there are miscellaneous typos and a few missing words, as well as some subject/verb disagreement throughout the text—particularly when using the word, ‘data.’

**Approval Status:**

 Based on the above assessment of the “Review of Health Research and Data on Racialised Groups: Implications for Addressing Racism and Racial Disparities in Public Health Practice and Policies in Europe” study protocol, the approval status given is: approved.

 Whilst there are still a few areas that could use improvement for clarity—particularly around operationalised definitions—overall the study protocol has been sufficiently revised to demonstrate its merits, relevance, timeliness, and importance to contribute to current scholarly dialogue around race and health disparities in the European context.  The current recommendations are minor in scope, and do not require an additional review for approval.

 My best wishes to the authors and research team as they undertake this important research project.

Is the study design appropriate for the research question?

Yes

Is the rationale for, and objectives of, the study clearly described?

Yes

Are sufficient details of the methods provided to allow replication by others?

Partly

Are the datasets clearly presented in a useable and accessible format?

Not applicable

Reviewer Expertise:

race, racialisation, racism(s), public health, health inequalities/inequities, human rights, cross-national comparison

I confirm that I have read this submission and believe that I have an appropriate level of expertise to confirm that it is of an acceptable scientific standard.

# Reviewer report: Reviewer response for version 1

**DOI:** 10.5256/f1000research.140910.r172212

**Published:** 2023-06-06

**Authors:** Chinelo L. Njaka

**Affiliations:** 1 Goldsmiths University of London, London, England, UK

**Introduction**

 Researchers, advocacy groups, and institutions have long advocated for the collection of disaggregated race data, emphasising its crucial role in promoting equality, combating discrimination, and informing evidence-based decision-making ( UN OHCHR 2018). Within the European context, some nations, influenced by various socio-historical factors, have been reluctant to gather demographic data that would highlight such disparities and provide insights into the experiences of different racialised and ethnic groups. This hesitance has led to ‘race-neutral’ policies, which have limited the availability of comprehensive and nuanced data on racialised inequalities, hindering efforts to understand fully the multiple factors that contribute to disparities and design targeted policies and interventions to move toward parity.

 “Review of Health Research and Data on Racialised Groups: Implications for Addressing Racism and Racial Disparities in Public Health Practice and Policies in Europe” offers a timely and exciting study protocol that focuses on assessing the available health data and research in France, the Netherlands, and Belgium, where limited attention has been given to racism and racialised inequalities in research and policy in comparable ways. The aim of the protocol is to highlight the importance of recognising racialisation and racism in public health practice and policies, and to emphasise the challenges in fully capturing and understanding racialised health disparities without consistent and explicit ways of identifying racialised groups within Europe. By systematically examining existing research, this protocol aims to uncover key insights that can inform efforts to address racism and mitigate racialised disparities in healthcare.

**Study Protocol Assessment**

 The significance and strength of this study protocol, as part of a larger project, lies in its potential to inform and shape future public health interventions and policies that aim to address racialised inequalities and inequities through advocating for a ‘race-conscious’ approach to European public health research. By critically examining the usage of race, ethnicity, and related terms in health-related research and analysing the collection and utilisation of data on racialised minority groups, the protocol provides valuable insights that can contribute to the future development of evidence-based guidance on the effective use of data and research with racialised groups, and promoting health equity across all racialised groups in Europe.

 The rationale for the protocol is adequately described, highlighting the need to address racism and racial disparities explicitly in public health practice and policies in Europe. The protocol acknowledges the significant health inequities experienced by racialised groups and aims to review existing health research and data to gain a comprehensive understanding of these disparities. The protocol also allows for the examination of the ways that researchers are conceptualising and operationalising racialised terminologies in order to identify disparities along racialised divisions in the first instance. The objectives of the study protocol are well-defined and aligned with the research rationale, focusing on conducting a systematic review of existing research and assessing the quality and accessibility of data on racialised groups. These objectives are relevant and essential for addressing the research scope and informing evidence-based interventions and policies.

 The study design appears appropriate for addressing the research objectives of reviewing health research and data on racialised groups in Europe. As outlined in the study protocol, a systematic review approach will be used to synthesise and analyse the available research and data using specific search parameters. This design allows for a comprehensive assessment of the determinants and consequences of racial disparities in public health outcomes. A particular strength of note is that, though this article is written in English, the scope of the protocol will analyse research additionally in Dutch and French, which reflects the languages used in the nations studied.

 The study protocol provides sufficient details of the methods employed in conducting the systematic review. The inclusion and exclusion criteria for selecting relevant studies are clearly described, which helps ensure the broad reproducibility of the review process. Additionally, the study protocol outlines the steps for data extraction, synthesis, and analysis, contributing to the replicability of the study. Due to the nature of the methods used, however, exact reproducibility to achieve the precise research and data identified is essentially impossible, due to the continuous addition of published research, search algorithms, and other factors outside of researchers’ control. This in no way reflects a deficit in the methods of the protocol.

**Areas for Improvement and Recommendations**

 Whilst the study protocol displays the commendable strengths outlined above, several areas could be enhanced to improve its effectiveness and impact.

 The main critique for the study protocol is the omission of bias consideration for the purposes of this research. It is imperative to acknowledge and address the biases that may arise during the research process. Providing statements of positionality seems to acknowledge this point, which is why it is surprising that there is no further elaboration on potential biases within the study protocol. Although the study is a critical review, the process still generates data and is critically assessed, so bias must be explicitly considered.

 Further to this point, there are some issues of potential bias present in the protocol that should be addressed. The protocol and broader research need to address the implicit use of Whiteness as the default and move away from binary categorisations of racial(ised) 'majority' and 'minority'. An intersectional lens should be applied to acknowledge the complexities of race and other intersecting social identities that affect the specific disparities seen among particular racialised groups, rather than amalgamating all racialised minorities implicitly into one disparate group. This approach will help highlight the diverse experiences and perspectives among racialised groups and avoid essentialisation or oversimplification. Additionally, ‘disparity’ is often used in the protocol to imply 'worse', ****which is not always the case. Disparity only refers to the difference or inequality between groups, which means that groups racialised as White are also part of the 'disparity equation'.

 The phrasing of data and research ‘on’ racialised groups can imply objectification, detachment, or othering of the communities of focus. Consider revising this language used throughout the protocol when discussing racialised and other minoritised or marginalised groups. The use of ‘with’ - also used in the protocol - better conveys the inclusivity advocated for throughout the protocol and wider research, and acknowledges the collaboration and partnership between researchers and the communities of focus.

 With acknowledgement that only the positionalities of the authors are explicitly described, the claim of a ‘racially diverse’ team may need to be re-evaluated to ensure that it accurately reflects meaningful diversity and avoids tokenism. If the positionality of the authors is reflective of the entire team, then what has been demonstrated is not racialised 'diversity'. ****Perhaps expand on what is meant by **‘**racially diverse **’ **to acknowledge the range of racialised and ethnic backgrounds represented in the research team, which would also strengthen the robustness of the methods that are informed by the positionalities of all of the team members involved.

 The research would benefit from a clearer distinction between the definitions of ‘race’ and ‘ethnicity’. As written, it is unclear how some of the characteristics used to define race are meaningfully distinct from the ‘cultural’ aspects used in the definition for ethnicity. The authors may also need to evaluate critically whether the non-phenotypic characteristics offered, such as clothing and speech, should be considered as aspects of race, as this can perpetuate reification and stereotypes of racialised groups (as warned against in CIHI 2022, cited in the protocol). If they do wish to retain these non-phenotypic characteristics as components of race their operational definition, the authors need to strengthen their argument by providing explicit rationale for the purposes of this protocol.

 The definitions offered for ‘inequality’ and ‘inequity’ in the protocol should be clearly referenced to establish the basis for these particular conceptualisations in this research and context. Depending on the field, area, or sector, these words have varying operational definitions, so the definitions offered are not necessarily universally accepted. Moreover, the definition of ‘inequity’ reads confusingly, and uses ‘equitable’ - a term not defined in the protocol - to explain it. The definitions could be revised to offer more straightforward explanations of the concepts; possibly by alternatively providing definitions in positive terms (i.e., ‘equality’ and ‘equity’) rather than (only) in negation.

 The protocol would also benefit from clarification and/or additional citations when referencing the definition for ‘xenophobia’. The protocol is mindful against conflation, however the definition given for xenophobia appears to conflate race and ethnicity. The use of terms such as ‘native’ and ‘host-country’ have been used uncritically, as though those who are racialised as minorities are necessarily ‘non-native’ and those who are not are necessarily ‘native’ and ‘hosts’. These terms can be problematised as reinforcing stereotypes or assumptions about individuals based on their racialised or ethnic backgrounds - as not all Europeans are part of a racialised majority, not all who are racialised as minorities are migrants, and especially with free movement within the European Union, not all who are racialised as part of a majority are from the location where they are living.

**Approval Status**

 Based on the above assessment of the “Review of Health Research and Data on Racialised Groups: Implications for Addressing Racism and Racial Disparities in Public Health Practice and Policies in Europe” study protocol, the approval status given is: 'approved with reservations'.

 Whilst the study protocol demonstrates several strengths and merits, there are areas that require improvement or clarification to enhance its effectiveness and impact. The strengths of the study protocol, including the clear rationale and objectives and appropriate study design, contribute to its overall value and support the 'approval with reservations' status. However, certain areas need attention to address reservations and ensure the quality and relevance of the protocol for this part of the research, as well as the wider research project. Recommendations have been provided regarding the need to consider and address potential biases and refine some of the language and key definitions used in the protocol. By implementing these recommendations, the study protocol can strengthen its potential for making a meaningful contribution to addressing racism and racialised disparities in public health practice and policies across Europe.

Is the study design appropriate for the research question?

Yes

Is the rationale for, and objectives of, the study clearly described?

Yes

Are sufficient details of the methods provided to allow replication by others?

Partly

Are the datasets clearly presented in a useable and accessible format?

Not applicable

Reviewer Expertise:

race, racialisation, racism(s), public health, health inequalities/inequities, human rights, cross-national comparison

I confirm that I have read this submission and believe that I have an appropriate level of expertise to confirm that it is of an acceptable scientific standard, however I have significant reservations, as outlined above.

# Response

**Published:** 2023-11-30

**Authors:** Marie Meudec

**Affiliations:** Institute of Tropical Medicine, Antwerp, Belgium

APPROVED WITH RESERVATIONS

 Introduction

 Researchers, advocacy groups, and institutions have long advocated for the collection of disaggregated race data, emphasising its crucial role in promoting equality, combating discrimination, and informing evidence-based decision-making (UN OHCHR 2018). Within the European context, some nations, influenced by various socio-historical factors, have been reluctant to gather demographic data that would highlight such disparities and provide insights into the experiences of different racialised and ethnic groups. This hesitance has led to ‘race-neutral’ policies, which have limited the availability of comprehensive and nuanced data on racialised inequalities, hindering efforts to understand fully the multiple factors that contribute to disparities and design targeted policies and interventions to move toward parity.

 “Review of Health Research and Data on Racialised Groups: Implications for Addressing Racism and Racial Disparities in Public Health Practice and Policies in Europe” offers a timely and exciting study protocol that focuses on assessing the available health data and research in France, the Netherlands, and Belgium, where limited attention has been given to racism and racialised inequalities in research and policy in comparable ways. The aim of the protocol is to highlight the importance of recognising racialisation and racism in public health practice and policies, and to emphasise the challenges in fully capturing and understanding racialised health disparities without consistent and explicit ways of identifying racialised groups within Europe. By systematically examining existing research, this protocol aims to uncover key insights that can inform efforts to address racism and mitigate racialised disparities in healthcare.

 Response: Summary by reviewer

 --

 Study Protocol Assessment

 The significance and strength of this study protocol, as part of a larger project, lies in its potential to inform and shape future public health interventions and policies that aim to address racialised inequalities and inequities through advocating for a ‘race-conscious’ approach to European public health research. By critically examining the usage of race, ethnicity, and related terms in health-related research and analysing the collection and utilisation of data on racialised minority groups, the protocol provides valuable insights that can contribute to the future development of evidence-based guidance on the effective use of data and research with racialised groups, and promoting health equity across all racialised groups in Europe.

 The rationale for the protocol is adequately described, highlighting the need to address racism and racial disparities explicitly in public health practice and policies in Europe. The protocol acknowledges the significant health inequities experienced by racialised groups and aims to review existing health research and data to gain a comprehensive understanding of these disparities. The protocol also allows for the examination of the ways that researchers are conceptualising and operationalising racialised terminologies in order to identify disparities along racialised divisions in the first instance. The objectives of the study protocol are well-defined and aligned with the research rationale, focusing on conducting a systematic review of existing research and assessing the quality and accessibility of data on racialised groups. These objectives are relevant and essential for addressing the research scope and informing evidence-based interventions and policies.

 The study design appears appropriate for addressing the research objectives of reviewing health research and data on racialised groups in Europe. As outlined in the study protocol, a systematic review approach will be used to synthesise and analyse the available research and data using specific search parameters. This design allows for a comprehensive assessment of the determinants and consequences of racial disparities in public health outcomes. A particular strength of note is that, though this article is written in English, the scope of the protocol will analyse research additionally in Dutch and French, which reflects the languages used in the nations studied.

 The study protocol provides sufficient details of the methods employed in conducting the systematic review. The inclusion and exclusion criteria for selecting relevant studies are clearly described, which helps ensure the broad reproducibility of the review process. Additionally, the study protocol outlines the steps for data extraction, synthesis, and analysis, contributing to the replicability of the study. Due to the nature of the methods used, however, exact reproducibility to achieve the precise research and data identified is essentially impossible, due to the continuous addition of published research, search algorithms, and other factors outside of researchers’ control. This in no way reflects a deficit in the methods of the protocol.

 Response: We thank the reviewer for their interest and positive remarks.

 --

 Areas for Improvement and Recommendations

 Whilst the study protocol displays the commendable strengths outlined above, several areas could be enhanced to improve its effectiveness and impact.

 The main critique for the study protocol is the omission of bias consideration for the purposes of this research. It is imperative to acknowledge and address the biases that may arise during the research process. Providing statements of positionality seems to acknowledge this point, which is why it is surprising that there is no further elaboration on potential biases within the study protocol. Although the study is a critical review, the process still generates data and is critically assessed, so bias must be explicitly considered.

 Response: We thank the reviewer for their comment. We acknowledge the importance of a thorough reflection on potential biases that may arise during the research.

 We added the following paragraph in the ‘Risk of Bias’-section :

 p.15: In addition to asserting our respective positionalities, we also identify several biases and limitations in our work. Firstly, our core team is small and represents only a margin of the diverse intersections of social identities that are present in society. Specifically, the core team is mostly trained in North American and European higher education, which implies that our own frames of reference are primarily Western. The categories and concepts we employ are therefore partial and limited, and inevitably bias our questions, methods and the interpretation and analysis of data both consciously and unconsciously. We are trying to mitigate this bias continually, for example by drawing from the field of Critical Race Theory, by gathering feedback from a larger and more diverse team of researchers, and by holding meetings with a range of stakeholders during future stages of the research (analysis, writing, and guideline development).

 Second, this research project focuses on ethnic and racial disparities in health, and thus centralises/emphasises race and racism. This may be a bias in that, by focusing on racial disparities, we temporarily sideline other criteria present within intersecting systems of oppression. We see this research as a first step in demonstrating the lack of a racial lens in health research, and we know that future research will need to take an intersectional lens as its starting point.

 A further bias may arise from the fact that we only have access to published results, and we do not have access to all the internal discussions or specific issues related to the use of specific terms or concepts over other options. As this analysis is something of a retrospective study, our analysis is based solely on the information that the authors decided to incorporate in their published manuscripts. This constitutes a bias in the sense that we may risk attributing certain intentions to the authors even though we cannot confirm this in this review. We regret the fact that, in general, authors of scientific articles in this field are not more explicit in justifying their use of a particular terminology.

 Another bias could stem from our personal and professional involvement with systemic racism in health. Given our respective positionalities and lived experiences, and given that we do not ultimately aim to achieve  objectivity or neutrality in this research, we would like to acknowledge that our judgment in analysing research papers can sometimes be harsh, especially after analysing several documents containing racial/ethnic proxies and euphemisms in the course of a single day. We try to mitigate this by holding weekly meetings with the core team, during which we share our emotions and feelings and discuss elements that emerge in the course of the research.

 Finally, this research project focuses on systemic racism in a European context. As such, health disparities are conceptualised in such a way that the white racial majority is seen as the benchmark of good health from which racially minoritised groups diverge. While we recognise that this is a simplified and binary way of analysing disparities, we also want to acknowledge that in doing so we are using whiteness as the default. We are aware of the need to decenter whiteness in research, and we urge the reader to be aware of this bias. Here are a few suggestions of intellectual traditions centering the voices of racialised groups that you can get inspiration from: Black Feminist Thought, Black Intellectual Tradition, Critical Race Theory, Indigenous methodologies, etc.

 --

 Further to this point, there are some issues of potential bias present in the protocol that should be addressed. The protocol and broader research need to address the implicit use of Whiteness as the default and move away from binary categorisations of racial(ised) 'majority' and 'minority'. An intersectional lens should be applied to acknowledge the complexities of race and other intersecting social identities that affect the specific disparities seen among particular racialised groups, rather than amalgamating all racialised minorities implicitly into one disparate group. This approach will help highlight the diverse experiences and perspectives among racialised groups and avoid essentialisation or oversimplification.

 Response:

 We have fallen into the trap of binary categorisations and the centering of Whiteness, and we thank the reviewer for pointing out this pertinent issue.

 Based on your comments, we have changed the term ‘racial minority groups’ for ‘racially minoritised groups’, and ‘minorities’ for ‘minoritised’ as suggested in the literature by Gunaratman (2013), Selvarajah et al. (2020), and Rai et al. (2022). We have added these references in the review protocol as well.

 We have changed Key definition 5 as follow:

 p6-7: 5. Racially minoritised groups : Refers to groups that are subject to racialisation and are also minoritised,  marginalised or underrepresented based on various characteristics such as skin colour, migration status, citizenship, religion, culture, language or geographic location . To emphasise the process of racialisation, some authors use either this wording (Milner and Jumbe, 2020 ; Rai et al., 2022) or “marginalised racial groups” (Barber, 2020). This quote from Selvarajah et al. (2020: 2-3) is particularly interesting for a reflection on the term ‘minoritised’ : “We recommend the term minoritised, which emphasises active processes, shifting beyond binary discussion of minority versus majority. We build on existing explanations to define minoritised, as ‘individuals and populations, including numerical majorities, whose collective cultural, economic, political and social power has been eroded through the targeting of identity in active processes that sustain structures of hegemony.’ Power is emphasised as central to racism and intersecting forms of discrimination. It highlights maintenance of structures which diminish minoritised people’s capability to lead healthy lives. It neither singles out nor creates groups, and adds more nuance than words like marginalised by connecting back to terms such as ethnic minority, thus acknowledging existing literature while resisting its coupling with dubious assumptions about ethnicity.

 And we have added Footnote 12:

 p.6: We would like to thank one of the reviewers of the first version of this protocol for their criticism, which helped us to refine our thinking on the risks of falling into binary categorisations and the centering of Whiteness.

 And this:

 p7: It is important to acknowledge the fact that racially minoritised groups are not homogenous groups, given the intersecting forms of oppression within minoritised groups. Although we clearly want to avoid any form of oversimplification, and we acknowledge the fact that health inequities cannot be reduced to race alone, we see a stark omission of research analysing racism in health in Europe ; therefore, we see this one-dimensional study as a first essential step to enable the development of more complex research using intersectional lens in the future.

 --

 Additionally, ‘disparity’ is often used in the protocol to imply 'worse', which is not always the case. Disparity only refers to the difference or inequality between groups, which means that groups racialised as White are also part of the 'disparity equation'.

 Response:

 We would like to thank the reviewer for pointing out this pertinent issue and by this helping us in communicating more clearly. We have revised our use of the word ‘disparity’ throughout the review and changed it where this was deemed necessary.

 For example:

 p2:  In many countries, there is a lack of national data systems using race/ethnicity data, which means that there is limited statistical evidence on health disparities between racially minoritised and majoritised groups.

 Identical corrections have been made throughout the text.

 Furthermore, definition 8 (health disparities) and 9 (racial health disparities) have been adapted accordingly:

 p7: Health disparities: Refers to a condition in which different social groups have different health outcomes. Generally, disadvantaged social groups such as the poor, racial/ethnic minoritised groups, women and other groups who have persistently experienced social disadvantage or discrimination systematically, experience worse health or greater health risks than more advantaged social groups (Braveman, 2004, 2007). When systemic barriers to good health are avoidable yet still remain, they are often referred to as 'health inequities'.

 p7-8: Racial health disparities: Refers to health disparities that occur between racially minoritised groups and the racially majoritised group. It describes the increased presence and severity of certain diseases, poorer health outcomes, and greater difficulty in obtaining healthcare services. Usually, it is the racially minoritised groups who are at a disadvantage compared to the racially majoritised one.

 --

 The phrasing of data and research ‘on’ racialised groups can imply objectification, detachment, or othering of the communities of focus. Consider revising this language used throughout the protocol when discussing racialised and other minoritised or marginalised groups. The use of ‘with’ - also used in the protocol - better conveys the inclusivity advocated for throughout the protocol and wider research, and acknowledges the collaboration and partnership between researchers and the communities of focus.

 Response:

 We thank the reviewer for their important observation and valuable suggestions.

 --

 Throughout the text, we have changed ‘research on racially minoritised groups’ into ‘research on/with racially minoritised groups’. In instances where the phrasing ‘data and research on racially minoritised groups’ is used, we have changed this into ‘data on and research on/with racially minoritised groups’. To be consistent, we have also modified this in the title of our review.

 We have also added the following footnote n.4:

 p2: The term ‘on/with’ is used deliberately. On the one hand, we want to acknowledge that in reality, much research is not done ‘with’ the communities it focuses on. On the other hand, however, we do not wish to reify and normalise the formulation ‘doing research on’ and the associated academic culture throughout our text. In addition, we only have access to the data that researchers have chosen to publish, so we do not always know whether the research is actually done ‘on’ or done ‘with’ the community in question. For all these reasons, using the formulation ‘on/with’ seems to be the most nuanced and correct way.

 --

 With acknowledgement that only the positionalities of the authors are explicitly described, the claim of a ‘racially diverse’ team may need to be re-evaluated to ensure that it accurately reflects meaningful diversity and avoids tokenism. If the positionality of the authors is reflective of the entire team, then what has been demonstrated is not racialised 'diversity'. Perhaps expand on what is meant by ‘racially diverse’ to acknowledge the range of racialised and ethnic backgrounds represented in the research team, which would also strengthen the robustness of the methods that are informed by the positionalities of all of the team members involved.

 Response:

 We thank the reviewer for their valuable comment.

 Indeed, the team expanded after the review protocol was drafted. In light of your comment and for clarity, we are removing the claim of "racial diversity" in the review protocol, and will further elaborate on the positionality of all team members, and their respective contributions to the project, when we publish the article presenting the results of the study.

 Therefore, we removed the claim as follow:

 p.2: This race-conscious approach forms the basis and the goal of this project, conducted by a racially diverse team (see below Review team / Positionality).

 For better clarity, we also added this section in the Description of the review team:

 p.14: Following ITM guidelines on authorship (2017), the core team has carried out the following tasks: 1) conception of the work; 2) design of the study and drafting of the review protocol.

 --

 The research would benefit from a clearer distinction between the definitions of ‘race’ and ‘ethnicity’. As written, it is unclear how some of the characteristics used to define race are meaningfully distinct from the ‘cultural’ aspects used in the definition for ethnicity. The authors may also need to evaluate critically whether the non-phenotypic characteristics offered, such as clothing and speech, should be considered as aspects of race, as this can perpetuate reification and stereotypes of racialised groups (as warned against in CIHI 2022, cited in the protocol). If they do wish to retain these non-phenotypic characteristics as components of race their operational definition, the authors need to strengthen their argument by providing explicit rationale for the purposes of this protocol.

 Response:

 We thank the reviewer for pointing out the confounding nature of these two definitions. We take this as an opportunity to further sharpen our definitions and our writing.

 The definition of ‘Race’ has been rewritten as such:

 p5: Race: Refers to socially and politically constructed perceptions of differences among people based on phenotypic characteristics such as skin colour. Although the sciences have been (and still are) heavily involved in the production of race and racial categorisations, there is no scientifically supported biological basis for racial categorisation. However, various societal actors construct races as real, which has a variety of detrimental implications for economic, political, social, and cultural life (CIHI, 2022).

 --

 The definitions offered for ‘inequality’ and ‘inequity’ in the protocol should be clearly referenced to establish the basis for these particular conceptualisations in this research and context. Depending on the field, area, or sector, these words have varying operational definitions, so the definitions offered are not necessarily universally accepted. Moreover, the definition of ‘inequity’ reads confusingly, and uses ‘equitable’ - a term not defined in the protocol - to explain it. The definitions could be revised to offer more straightforward explanations of the concepts; possibly by alternatively providing definitions in positive terms (i.e., ‘equality’ and ‘equity’) rather than (only) in negation.

 Response:

 We thank the reviewer for pointing this out and have revised the definitions. We have brought in a more specific focus on health inequality and health inequity, rather than attempting to define the concepts in general (separately from health). This will help us make the definitions less abstract and more relevant and to the point. Therefore, we have removed the definitions of Inequality and Inequity. In addition, we have merged the definition of health inequality with the definition of health disparities:

 p7: Health disparities or health inequalities: Refers to a condition in which different social groups have different health outcomes. Generally, disadvantaged social groups such as the poor, racially/ethnic minoritised groups, women and other groups who have persistently experienced social disadvantage or discrimination systematically, experience worse health or greater health risks than more advantaged social groups (Braveman, 2004, 2007). When systemic barriers to good health are avoidable yet still remain, they are often referred to as 'health inequities'.

 p7: Health equity: Refers to the absence of differences in health associated with social disadvantages that are modifiable, and considered unfair. This means everyone has a fair chance to reach their full health potential without being disadvantaged by social, economic and environmental conditions (CIHI, 2022 ; NCCDH, 2014).

 --

 The protocol would also benefit from clarification and/or additional citations when referencing the definition for ‘xenophobia’. The protocol is mindful against conflation, however the definition given for xenophobia appears to conflate race and ethnicity. The use of terms such as ‘native’ and ‘host-country’ have been used uncritically, as though those who are racialised as minorities are necessarily ‘non-native’ and those who are not are necessarily ‘native’ and ‘hosts’. These terms can be problematised as reinforcing stereotypes or assumptions about individuals based on their racialised or ethnic backgrounds - as not all Europeans are part of a racialised majority, not all who are racialised as minorities are migrants, and especially with free movement within the European Union, not all who are racialised as part of a majority are from the location where they are living.

 Response:

 Once again, we thank the reviewer for their comment. In fact, their remark is the point we are trying to make in this paragraph discussing the definition of xenophobia and especially discussing how it is distinct from racism. As this is not understood as such, we have rewritten the definition to make it more to the point and the message clearer.

 p9: Xenophobia: “Attitudes, prejudices and behaviour that reject, exclude and often vilify persons, based on the perception that they are outsiders or foreigners to the community, society or national identity.” (European Commission, Migration and Home Affairs) As such, xenophobia needs to be distinguished from racism, which concerns (systemic) acts of discrimination towards someone based on their perceived affiliation to a racially minoritised group, regardless whether this person is seen as a foreigner or not (Suleman et al., 2018: 2). This distinction is important, as the mechanisms and outcomes of both forms of discrimination are different and can affect different groups.

 --

 Approval Status

 Based on the above assessment of the “Review of Health Research and Data on Racialised Groups: Implications for Addressing Racism and Racial Disparities in Public Health Practice and Policies in Europe” study protocol, the approval status given is: 'approved with reservations'.

 Whilst the study protocol demonstrates several strengths and merits, there are areas that require improvement or clarification to enhance its effectiveness and impact. The strengths of the study protocol, including the clear rationale and objectives and appropriate study design, contribute to its overall value and support the 'approval with reservations' status. However, certain areas need attention to address reservations and ensure the quality and relevance of the protocol for this part of the research, as well as the wider research project. Recommendations have been provided regarding the need to consider and address potential biases and refine some of the language and key definitions used in the protocol. By implementing these recommendations, the study protocol can strengthen its potential for making a meaningful contribution to addressing racism and racialised disparities in public health practice and policies across Europe.

 Response:

 We thank the reviewer for their thorough feedback and helpful suggestions. We will share our results with you when they are ready.

# Reviewer report: Reviewer response for version 1

**DOI:** 10.5256/f1000research.140910.r164519

**Published:** 2023-05-10

**Authors:** Elie Azria

**Affiliations:** 1 INSERM, Université de Paris, Paris, France

I would like to thank the editorial team for allowing me to review this very interesting project which I found very instructive. Research on racialized groups is a major issue in informing policies to reduce health inequalities.

 The authors present the protocol for a systematic review of research on the health of racialized groups. The aim of this systematic review is not to assess the health of these groups through health indicators or to measure health inequalities that might exist with a reference group, but to produce a critical analysis of this research and the concepts it mobilises, particularly in the categorisation of social groups. Based on this critical analysis, the authors aim to promote a race-conscious approach to health research and to strengthen the use of health research data in Europe.

 The authors start from the assumption, which is unfortunately poorly supported by arguments and references, that research on racialized groups in Europe is mostly insufficient and inadequate. This assertion should be further documented.

 While this is an extremely interesting approach, the authors argue that exposure variables other than those that can identify racialized groups are proxies or euphemisms. I find this problematic in that these variables can be used to good effect for specific purposes. For example, using ethno-racial variables to characterise a group when the research question concerns the study of health inequalities between migrants and native-born patients would be misguided. Place of birth is much more interesting here and is in no way a proxy. Without denying the use of proxies in many research studies, thinking that this is systematically the case when the exposure variables do not allow for the identification of a racialized group risks putting this analysis on the wrong track.

 Furthermore, the choice not to assess the risk of bias seems to me unfortunate in that reporting on the quality of research on this issue could add to the project, even if the study of outcomes is not the objective of this review.

 Finally, this protocol should be registered on the Prospero platform, which leads to the discussion of the relevance of another publication.

 I wish the authors of this interesting project great success.

Is the study design appropriate for the research question?

Partly

Is the rationale for, and objectives of, the study clearly described?

Yes

Are sufficient details of the methods provided to allow replication by others?

Yes

Are the datasets clearly presented in a useable and accessible format?

Not applicable

Reviewer Expertise:

Obstetrics & gynecology; epidemiology; health inequalities

I confirm that I have read this submission and believe that I have an appropriate level of expertise to confirm that it is of an acceptable scientific standard, however I have significant reservations, as outlined above.

# Response

**Published:** 2023-11-30

**Authors:** Marie Meudec

**Affiliations:** Institute of Tropical Medicine, Antwerp, Belgium

APPROVED WITH RESERVATIONS

 I would like to thank the editorial team for allowing me to review this very interesting project which I found very instructive. Research on racialized groups is a major issue in informing policies to reduce health inequalities.

 The authors present the protocol for a systematic review of research on the health of racialized groups. The aim of this systematic review is not to assess the health of these groups through health indicators or to measure health inequalities that might exist with a reference group, but to produce a critical analysis of this research and the concepts it mobilises, particularly in the categorisation of social groups. Based on this critical analysis, the authors aim to promote a race-conscious approach to health research and to strengthen the use of health research data in Europe.

 Response: We thank the reviewer for their interest.

 The authors start from the assumption, which is unfortunately poorly supported by arguments and references, that research on racialized groups in Europe is mostly insufficient and inadequate. This assertion should be further documented.   

 Response: These are indeed our hypotheses, but the scale of the problem needs to be demonstrated in our study. We wanted to highlight certain trends in the field of health research, and in particular the fact that the categories and variables used in a certain number of publications (out of a total of 700 articles) are not necessarily in line with the research objectives or with the interpretations made of the results. Although our research has some limitations (we study mostly published articles in scientific journals, and we know that some research is not published (yet)), our objective is indeed to prove our hypotheses. Based on the results of our review, we will be able to give a better idea of the research landscape on/with racially minoritised groups in these 3 countries.

 For better clarification, we have added this in a revised version of our review protocol:

 (Page numbers are those of the version with track changes)

 p.2: Yet, we start from the assumption that, in many countries across Europe, there is often inappropriate, inadequate, or insufficient use and deployment of data and research* on racially minoritised groups. The reasons for this assumption can be grouped into two main categories. One is the continuous emergence of biologically or genetically based race research which is often linked to scientific racism (Roberts, 2011a, 2011b; Saini, 2019).

 While this is an extremely interesting approach, the authors argue that exposure variables other than those that can identify racialized groups are proxies or euphemisms. I find this problematic in that these variables can be used to good effect for specific purposes. For example, using ethno-racial variables to characterise a group when the research question concerns the study of health inequalities between migrants and native-born patients would be misguided. Place of birth is much more interesting here and is in no way a proxy. Without denying the use of proxies in many research studies, thinking that this is systematically the case when the exposure variables do not allow for the identification of a racialized group risks putting this analysis on the wrong track.

 Response: We thank the reviewer for their comment and fully agree with their remarks. We agree that concepts are not inherently a proxy or a euphemism, that this is relative to the research question and that different variables can be useful for different purposes.

 We take this comment as an encouragement to explain our understanding of proxies and euphemisms in a more clear and nuanced way. We do this by rewriting Key definition 3:

 p5: Racial and ethnic euphemisms and proxies: A word or expression used inappropriately or inadequately to designate and describe racially minoritised groups. We understand concepts as racial and ethnic euphemisms and proxies when there is a clear mismatch between 1) the used concepts or variables and 2) the aim of the research or the interpretation of the results. This includes the use of terms which are related to concepts like migration, religion, language, origin, postcode (= place-based discrimination), citizenship, nationality, and culture. Note however that the concepts mentioned above are not inherently proxies or euphemisms for race and ethnicity , and can be of adequate use depending on the topic of research.

 Furthermore, the choice not to assess the risk of bias seems to me unfortunate in that reporting on the quality of research on this issue could add to the project, even if the study of outcomes is not the objective of this review.

 Response: We thank the reviewer for their comment. Because we do not plan to study the outcomes of the studies, we have decided not to make assessments for the risk of bias by study. We understand that this could have benefited the project. However, although we do not use a tool to systematically assess risk of bias, we believe that we largely account for the quality of the research in our critical analysis by examining the concepts and terms used, the types of data, and the methodologies employed in each study. We also added some information in the Risk of bias section.

 Finally, this protocol should be registered on the Prospero platform, which leads to the discussion of the relevance of another publication.

 Response: We thank the reviewer for their suggestion. On the website of the Prospero platform the following is stated: “from 1st October 2019, we will only accept reviews provided that data extraction has not yet started.” We have already taken significant steps in the data extraction, thus we are not eligible anymore to register on the Prospero platform at this stage of the research.

 I wish the authors of this interesting project great success.

 Response: Thank you very much! We will share our results with you when they are ready.
